# Vaccines and Vaccination: Feature Papers

**DOI:** 10.3390/vaccines13070720

**Published:** 2025-07-01

**Authors:** Pedro Plans-Rubió

**Affiliations:** College of Physicians of Barcelona, 08017 Barcelona, Spain; pedro.plans@yahoo.es

This Special Issue, entitled “Vaccines and vaccination: Feature Papers”, included articles that addressed various issues related to vaccines and vaccination, including studies assessing interventions to increase vaccination coverage [1], studies assessing vaccination coverage indicators in World Health Organization (WHO) regions [2], studies assessing vaccination hesitancy and vaccination acceptance [3,4,5,6], economic studies of cancer vaccines [7], reviews regarding the development of new vaccines [8,9], and reviews of vaccine-preventable diseases [10].

The article by Gerin et al. [1] presented the results of a study assessing the effectiveness of a training course for health professionals that aimed to enhance vaccination coverage among people living with HIV (PLHIV) in Brazil. The study found that the training course resulted in greater vaccination coverage for the 13- and 23-valent pneumococcal vaccines, human papilloma virus vaccine, meningococcal C vaccine, measles–mumps–rubella vaccine, and yellow fever vaccine. The training course was effective due to the fact that recommendation by a health professional is one of the factors influencing vaccination acceptance [11,12].

The article by Plans-Rubió [2] assessed the coverage of measles vaccination with zero, one and two doses of vaccine and the anti-measles herd immunity levels in World Health Organization (WHO) regions in 2023; and the variation from 2019 to 2023 for measles vaccination coverage and anti-measles herd immunity-related indicators. To achieve the Immunization Agenda objective of eliminating measles in at least five of the six WHO regions by 2030 (IA2030), it is necessary to increase the two-dose vaccination coverage to ≥95% in all countries and WHO regions, and to reduce the number of children who have not received at least one measles vaccine (zero-dose measles children) by 50% from 2019 to 2030 [13,14,15]. However, this study found that the global two-dose measles vaccination coverage decreased by 3.7%, the global zero-dose measles vaccination coverage increased by 7.8, and the number of countries with ≥95% two-dose measles vaccination coverage decreased by 39.6% from 2019 to 2023. This study concluded that measles vaccination programs should therefore be improved in all WHO regions in order to eradicate measles worldwide.

Four articles published in this Special Issue were focused on vaccination acceptance and vaccination hesitancy [3,4,5,6]. Vaccine hesitance is defined as a delay in the acceptance or refusal of vaccines despite the availability of vaccine services [16,17]. Vaccine hesitancy is a complex issue influenced by factors such as misinformation, complacency, and a lack of confidence in vaccines and vaccination services [18,19,20]. Christodoulakis et al. [3] developed a scoping review study to assess the hesitancy towards COVID-19 booster doses among healthcare workers worldwide. The study found vaccine hesitancy rates of 19.7% to 66.5% in Asia, 27% to 46.1% in Africa, and 14% to 60.2% in Europe. The vaccine hesitancy rates ranged from 12.8% to 43.7% among physicians, 26% to 37% among nurses, and 26% to 34.6% among pharmacists. The study concluded that future pandemic vaccination programs should develop activities to improve vaccination rates among healthcare workers.

The article by Vicente-Alcaide et al. [4] assessed the acceptance of COVID-19 vaccination among Spanish prisoners and prison workers, finding that 88.72% of prisoners and prison workers agreed to be vaccinated and 89.64% would recommend the vaccine to others. In addition, 89% of prisoners and prison workers believed that the benefits of COVID-19 vaccination were greater than its potential adverse effects.

The article by De Vito [5] presents the results of a narrative review regarding the vaccinations available for adults living with HIV. This study found that vaccinations were strongly recommended among adults living with HIV, although data on immunogenicity, tolerability, and clinical efficacy were limited. This article concluded that clinicians should collect the history of vaccinations in all new patients with HIV and assess their antibody levels against different vaccine-preventable pathogens, especially in patients with low CD4 numbers. 

The article by Limbu and Gautam [6] presents the results of a systematic review assessing the relationships between Health Belief Model (HBM) constructs and COVID-19 vaccination intention. This study found that perceived benefits, perceived barriers, and cues to action were the three most frequent predictors for primary and booster COVID-19 vaccination.

One of the articles included in this Special Issue focused on the economic analysis of a new cancer vaccine. Novakova et al. [7] evaluated the financial feasibility of introducing a peptide-based neoantigen cancer vaccine (NCV) for the treatment of patients with triple-negative breast cancer (TNBC). The survival rate at five years for patients with metastatic TNBC is 10%, while that for other metastatic breast cancer subtypes is 30%, and they have limited treatment options [21,22]. The study proposes that a neoantigen cancer vaccine could enhance T-cell responses independently of genetic factors, unlike approved immunotherapies for TNBC. In the UK, the conventional treatment costs per patient with TNBC range from GBP 2200 to GBP 54,000, and the total costs of treating TNBC patients reached GBP 230 million in 2024. The incremental cost-effectiveness ratio in terms of cost per life year for the quality-adjusted life year gained (QALY) of approved TNBC therapies was GBP 52,000 for atezolizumab, GBP 34,000 for pembrolizumab and GBP 38,000 for chemotherapy. The incremental cost-effectiveness ratio for a NCV vaccine (11 doses) was GBP 2200 in the best-case scenario, where the NCV was assumed to be administered at cost with a decentralized approach and zero intermediary supply chain. In contrast, the incremental cost-effectiveness ratio was GBP 55,000 in the worst-case scenario, where the NCV was produced by a laboratory that aimed to recover the research and development costs. The cost-effectiveness ratios of the NCV were lower and greater than the NICE willingness-to pay threshold of GBP 50,000 in the best-case scenario and the worse-case scenario, respectively.

The article by Choi et al. [8] assessed the efficacy of a new vaccine against *Clostridium botulinum* Neurotoxin Types A and B in an animal model (mice). The vaccine was generated by combining the HCC domains of botulinum neurotoxin type A and type B in *Escherichia coli* to produce a recombinant protein (rHCCB-L-HCCArHCcB) that inhibits their receptor binding. This study found that mice immunized with the anti-neurotoxin vaccine had significantly greater levels of antibodies than mice immunized with alum alone, showing that this vaccine was effective in protecting against lethal levels of neurotoxins of type A and type B.

The article by Onnocks et al. [9] presents a review concerning the potential use of attenuated and/or genetically modified oncolytic viruses (OV) for cancer therapy. Tumor antigen-presenting vaccines can be based on peptides, DNA, and dendritic cells as antigen-presenting cells. Oncolytic viruses are non-pathogenic viruses that can initiate post-oncolytic anti-cancer immunity by infecting cancer cells and cause oncolysis [23].

The article by Dhawan et al. [10] presents a review addressing the influence of regulatory T-cells (Tregs) on the prognosis of COVID-19. Decreased levels of Tregs among COVID-19 patients can be associated with lower inflammatory inhibition, which is associated with a worse COVID-19 prognosis. The severity of COVID-19 is also associated with abnormalities in the Tregs phenotype, including a reduction in the expression of FoxP3, IL-10 and TGF-beta [24]. This study shows the need to maintain COVID-19 vaccination among vulnerable populations who are at a greater risk of COVID-19 complications.

The articles published in this Special Issue reveal that the success of vaccination programs can depend on multiple factors, including the development and production of effective and cost-effective vaccines, vaccination acceptance and hesitancy, and the effective and efficient implementation of vaccination programs.

## References

[1] Gerin L., Gir E., Neves L.A.d.S., Passos L.M.R., Kfouri R.d.Á., Spire B., Reis R.K. (2024). Vaccination Coverage of People Living with HIV: Before and after Interventional Action. Vaccines.

[2] Plans-Rubió P. (2025). Measles Vaccination Coverage and Anti-Measles Herd Immunity Levels in the World and WHO Regions Worsened from 2019 to 2023. Vaccines.

[3] Christodoulakis A., Bouloukaki I., Aravantinou-Karlatou A., Zografakis-Sfakianakis M., Tsiligianni I. (2024). Vaccine Hesitancy and Associated Factors Amongst Health Professionals: A Scoping Review of the Published Literature. Vaccines.

[4] Vicente-Alcalde N., Sferle S.M., Franco-Paredes C., Tuells J. (2023). Acceptance of the COVID-19 Vaccine by Prisoners and Staff in Spanish Prisons. Vaccines.

[5] De Vito A., Colpani A., Trunfio M., Fiore V., Moi G., Fois M., Leoni N., Ruiu S., Babudieri S., Calcagno A. (2023). Living with HIV and Getting Vaccinated: A Narrative Review. Vaccines.

[6] Limbu Y.B., Gautam R.K. (2023). How Well the Constructs of Health Belief Model Predict Vaccination Intention: A Systematic Review on COVID-19 Primary Series and Booster Vaccines. Vaccines.

[7] Novakova A., Morris S.A., Vaiarelli L., Frank S. (2025). Manufacturing and Financial Evaluation of Peptide-Based Neoantigen Cancer Vaccines for Triple-Negative Breast Cancer in the United Kingdom: Opportunities and Challenges. Vaccines.

[8] Choi E.-S., Pyo S.-W., Kim S.-H., Jeon J.-H., Rhie G.-E., Yun M.-R., Yi H., Chung Y.-S. (2025). Development of a Recombinant Fusion Vaccine Candidate Against Lethal *Clostridium botulinum* Neurotoxin Types A and B. Vaccines.

[9] Onnockx S., Baldo A., Pauwels K. (2023). Oncolytic Viruses: An Inventory of Shedding Data from Clinical Trials and Elements for the Environmental Risk Assessment. Vaccines.

[10] Dhawan M., Rabaan A.A., Alwarthan S., Alhajri M., Halwani M.A., Alshengeti A., Najim M.A., Alwashmi A.S.S., Alshehri A.A., Alshamrani S.A. (2023). Regulatory T Cells (Tregs) and COVID-19: Unveiling the Mechanisms, and Therapeutic Potentialities with a Special Focus on Long COVID. Vaccines.

[11] Wennekes M.D., Almási T., Eilers R., Mezei F., Petykó Z.I., Timen A., Vokó Z., VITAL Consortium (2024). Effectiveness of educational interventions for healthcare workers on vaccination dialogue with older adults: A systematic review. Arch. Public Health.

[12] Gallup (2018). Wellcome Global Monitor. https://wellcome.org/sites/default/files/wellcome-global-monitor-2018.pdf.

[13] WHO/UNICEF Immunization Agenda 2030: A Global Strategy to Leave No One Behind. https://www.who.int/publications/m/item/immunization-agenda-2030-a-global-strategy-to-leave-no-one-behind#:~:text=The%20Immunization%20Agenda%202030%20(IA2030,opportunities%20to%20meet%20those%20challenges.

[14] WHO-UNICEF Progress and Challenges with Achieving Universal Immunization Coverage. https://www.who.int/publications/m/item/progress-and-challenges-with-achievinguniversal-immunization-coverage.

[15] World Health Organization (WHO) (2020). Measles and Rubella Strategic Framework 2021–2030.

[16] Mac Donald N.E., SAGE Working Group on Vaccine Hesitancy (2015). Vaccine hesitancy: Definition, scope and determinants. Vaccine.

[17] European Centre for Disease Prevention and Control (ECDC) (2017). Catalogue of Interventions Addressing Vaccine Hesitancy.

[18] Dubé E., Laberge C., Guay M., Bramadat P., Roy R., Bettinger J. (2013). Vaccine hesitancy: An overview. Hum. Vaccines Immunother..

[19] Larson H.J., Gakidou E., Murray C.J.L. (2022). The Vaccine-Hesitant Moment. N. Engl. J. Med..

[20] The Lancet Child Adolescent Health (2019). Vaccine hesitancy: A generation at risk. Lancet Child. Adolesc. Health.

[21] Hsu J.Y., Chang C.J., Cheng J.S. (2022). Survival, treatment regimens and medical costs of women newly diagnosed with metastatic triple-negative breast cancer. Sci. Rep..

[22] Dieci M.V., Tsvetkova V., Orvieto E., Piacentini F., Ficarra G., Griguolo G., Miglietta F., Giarratano T., Omarini C., Bonaguro S. (2018). Immune characterization of breast cancer metastases: Prognostic implications. Breast Cancer Res..

[23] Schirrmacher V. (2020). Cancer Vaccines and Oncolytic Viruses Exert Profoundly Lower Side Effects in Cancer Patients than Other Systemic Therapies: A Comparative Analysis. Biomedicines.

[24] Grau-Expósito J., Sánchez-Gaona N., Massana N., Suppi M., Astorga-Gamaza A., Perea D., Rosado J., Falcó A., Kirkegaard C., Torrella A. (2021). Peripheral and Lung Resident Memory T Cell Responses against SARS-CoV-2. Nat. Commun..

