# Correction: Plans-Rubió, P. Vaccines and Vaccination: Feature Papers. *Vaccines* 2025, *13*, 720

**DOI:** 10.3390/vaccines13101005

**Published:** 2025-09-25

**Authors:** Pedro Plans-Rubió

**Affiliations:** College of Physicians of Barcelona, 08017 Barcelona, Spain; pedro.plans@yahoo.es

The author would like to make the following correction to this publication [1].

In the original publication, one article from the Special Issue was inadvertently omitted:

Limbu, Y.B.; Gautam, R.K. How Well the Constructs of Health Belief Model Predict Vaccination Intention: A Systematic Review on COVID-19 Primary Series and Booster Vaccines. *Vaccines* **2023**, *11*, 816.

The author would like to insert this article as the sixth reference in [1], together with the original references [3–5], therefore changing the sentence “studies assessing vaccination hesitancy and vaccination acceptance [3–5]” to “studies assessing vaccination hesitancy and vaccination acceptance [3–6]” in the first paragraph. Following this change, all subsequent references cited in [1] will be reordered.

In the fourth paragraph, the first sentence is changed from “Three articles published in this Special Issue were focused on vaccination acceptance and vaccination hesitancy [3–5]” to “Four articles published in this Special Issue were focused on vaccination acceptance and vaccination hesitancy [3–6]”.

The following paragraph should be inserted as the seventh paragraph:

“The article by Limbu and Gautam [6] presents the results of a systematic review assessing the relationships between Health Belief Model (HBM) constructs and COVID-19 vaccination intention. This study found that perceived benefits, perceived barriers, and cues to action were the three most frequent predictors for primary and booster COVID-19 vaccination.”

The author states that the scientific conclusions are unaffected. This correction was approved by the Academic Editor. The original publication has also been updated.
